# Acquisition of Sexually Transmitted Infections among Women Using a Variety of Contraceptive Options: A prospective Study among High‐risk African Women

**DOI:** 10.1002/jia2.25257

**Published:** 2019-02-28

**Authors:** Flavia Matovu Kiweewa, Elizabeth Brown, Anu Mishra, Gonasagrie Nair, Thesla Palanee‐Phillips, Nyaradzo Mgodi, Clemensia Nakabiito, Nahida Chakhtoura, Sharon L Hillier, Jared M Baeten, the MTN‐020/ASPIRE Study Team, Lydia Soto‐Torres, Katie Schwartz, Bonus Makanani, Francis Martinson, Linda‐Gail Bekker, Vaneshree Govender, Felix Mhlanga

**Affiliations:** ^1^ Makerere University ‐ Johns Hopkins University Research Collaboration Kampala Uganda; ^2^ Makerere University School of Public Health Kampala Uganda; ^3^ Fred Hutchinson Cancer Research Center Seattle WA USA; ^4^ University of Washington Seattle WA USA; ^5^ Emavundleni Research Centre CapeTown Republic of South Africa; ^6^ Wits Reproductive Health and HIV Institute University of the Witwatersrand Johannesburg Republic of South Africa; ^7^ University of Zimbabwe College of Health Sciences Clinical Trials Research Centre Harare Zimbabwe; ^8^ National Institute of Child Health and Human Development/National Institute of Health Bethesda MD USA; ^9^ University of Pittsburgh Pittsburgh PA USA

**Keywords:** hormonal contraception, sexually transmitted diseases, chlamydia, gonorrhoea, trichomoniasis

## Abstract

**Introduction:**

In many African settings, women concurrently face substantial risk of human immunodeficiency virus type 1 (HIV‐1) infection, sexually transmitted infections (STIs) and unintended pregnancies. Few studies have evaluated STI risk among users of hormonal implants and copper intrauterine devices (IUDs) although these long‐acting reversible contraceptive methods are being promoted widely because of their benefits. Within a prospective study of women at risk for HIV‐1, we compared the risk of acquisition of *Chlamydia trachomatis, Neisseria gonorrhoeae* and *Trichomonas vaginalis* among women using different contraceptive methods.

**Methods:**

MTN‐020/ASPIRE was a randomized trial of the dapivirine vaginal ring for HIV‐1 prevention among 2629 women aged 18 to 45 years from Malawi, South Africa, Uganda and Zimbabwe, of whom 2264 used copper IUDs or progestin‐based injectables or implants during follow‐up. Screening for the above STIs occurred semi‐annually.

**Results:**

Over 3440 person‐years of follow‐up, 408 cases of *C. trachomatis* (incidence 11.86/100 person‐years), 196 of *N. gonorrhoeae* (5.70/100 person‐years) and 213 cases of *T. vaginalis* (6.19/100 person‐years) were detected. *C. trachomatis* and *N. gonorrhoeae* incidence were not significantly different across contraceptive methods. *T. vaginalis* incidence was significantly higher for copper IUD users compared to depot medroxyprogesterone acetate (DMPA), implant and norethisterone enanthate users.

**Conclusion:**

Among African women at high HIV‐1 risk, STIs were common. Risk of cervical infections did not differ across contraceptive methods. Significantly higher rates of *T. vaginalis* were observed among progestin‐based methods compared to copper IUD users. Overall, these findings call for more intensive routine screening for STIs, and they support current World Health Organization guidance that women should have a wide range of contraceptive options.

## 1 Introduction

The World Health Organization (WHO) estimates that 357 million adults acquire one of four curable sexually transmitted infections (STIs: *Chlamydia trachomatis*,* Neisseria gonorrhoeae*,* Treponema pallidum* and *Trichomonas vaginalis*) each year 1, with the highest burden falling disproportionately on low‐income countries 2. In these countries, STIs are the second leading cause of lost healthy life‐years in women of reproductive age because of their adverse effects on reproductive health 2, 3. In addition, STIs increase the risk of HIV‐1 acquisition and transmission 4, 5, 6, 7, 8, 9, providing further incentive to understand factors increasing STI susceptibility.

Hormonal contraceptives, including combined oral contraceptive pills, injectable depot medroxyprogesterone acetate (DMPA) and norethisterone enanthate (NET‐EN), and hormonal‐containing implants, are among the most widely used family planning methods in many countries where the burden of both HIV‐1 and other STIs is highest 10. Although the association between HIV‐1 acquisition and the use of hormonal contraception has been extensively studied 4, 5, 6, 7, 8, 9, 11, 12, with the bulk of studies suggesting modestly elevated HIV‐1 risk for DMPA users, relatively fewer studies have examined STI acquisition among different groups of contraceptive users. Prior prospective studies examining the association between use of certain hormonal contraceptives and the risk of STIs have reported mixed findings 9, 13, 14, 15, 16, 17, 18, with some reporting a statistically significantly higher incidence of certain STIs among users of specific contraceptive methods 14, 19 while others found no association 13, 15, 16, 17. Several of these studies had important methodological shortcomings, including insensitive STI diagnostics, wide variability in exposure definitions, and limited control for potential confounding factors particularly sexual behaviour which is closely linked to contraceptive choice. Very few prospective studies have evaluated STI risk among users of hormonal implants 14 and copper intrauterine devices (IUDs) 20, although these long‐acting reversible contraceptive methods are increasingly being used worldwide. In addition, nearly all studies have compared STI acquisition for users of contraceptive methods compared to women not using contraception, although there are likely substantial measurable and unmeasurable differences between women who choose to use versus not use contraception. For this reason, the more relevant risk calculus for women choosing a contraceptive method is STI risk between different methods, not against no use. We therefore compared the risk of STI acquisition among women using different contraceptive methods within a prospective study of a vaginal ring for HIV‐1 prevention (MTN‐020/ASPIRE) 21.

## 2 Methods

### 2.1 Study design and participants

We analysed prospective data from the ASPIRE study, a randomized, double‐blind, placebo‐controlled trial of a monthly vaginal ring containing dapivirine, a non‐nucleoside HIV‐1 reverse transcriptase inhibitor, among 2629 women aged 18 to 45 years. Women were enrolled at sites in Malawi (n = 272), South Africa (n = 1426), Uganda (n = 253) and Zimbabwe (n = 678) from August 2012 to June 2014, and followed until June 2015 21. Eligible women were randomized 1:1 to either a silicone elastomer vaginal matrix ring containing 25 mg of dapivirine or a placebo vaginal ring, to be worn continuously and changed monthly. Women were eligible for study participation if HIV‐1 seronegative, sexually active (defined as at least one sexual act in the three months prior to screening), non‐pregnant, had no untreated curable genitourinary infection and were willing to use effective contraception (i.e. a hormonal method, intrauterine device or surgical sterilization). Potential participants with curable STIs and other genitourinary infections (except for asymptomatic candidiasis) diagnosed at screening were eligible for enrolment after completing treatment per World Health Organization guidelines 22. No test of cure was performed per the guidelines. Women who were breastfeeding or had abnormal renal, hepatic or haematologic function were excluded. Compared to placebo, the dapivirine vaginal ring reduced HIV‐1 acquisition risk by 27% overall and by >50% in subgroups with evidence of greater adherence 21.

### 2.2 Procedures

Women attended monthly visits for study product provision, completion of standardized questionnaires regarding sexual behaviour, and testing for pregnancy and HIV‐1 infection status. Contraceptive use was documented monthly, and all sites provided multiple methods on‐site; for women who received contraception from off‐site clinics, current method was transcribed from family planning clinic cards. All participants received a package of STI prevention services, including risk reduction counselling, treatment of STIs for participants and partners, and free condoms.

### 2.3 Detection of *N. gonorrhoeae*,* C. trachomatis* and *T. vaginalis*

Screening for *C. trachomatis*,* N. gonorrhoeae* and *T. vaginalis* was performed at baseline, every six months, at the final study visit, and additionally when clinically indicated (e.g. symptoms, or partner symptoms). *C. trachomatis* and *N. gonorrhoeae* were detected in urine using Becton Dickinson ProbeTec ET CT/GC Amplified DNA Assay, and *T. vaginalis* was detected on vaginal swabs using a rapid Trichomonas OSOM BV BLUE test.

### 2.4 Statistical analysis

Data were analysed using R (R Foundation for Statistical Computing; Vienna, Austria). Means (standard deviations) and medians (inter‐quartile ranges) were used to summarize continuous data. We compared risk of STI acquisition among women using different contraceptive methods, specifically, DMPA, and NET‐EN, and hormonal‐containing implants, to copper IUD users. The at‐risk population are women who were exposed to any of these contraceptive methods during the specified visit interval. We defined the primary method of contraception to be the method of contraceptive that a woman was currently using and had been on for the longest duration in cases of concurrent contraceptive exposure. Outcomes were a positive test result for *C. trachomatis*,* N. gonorrhoeae* or *T. vaginalis*, each analysed separately. Because STIs were treated as part of study procedures, we defined an incident infection as any positive screening test, and a woman could have multiple incident infections in the analysis. Participants were excluded from the analysis if not using any of the following methods of interest: copper IUD, Implant, DMPA, or NET‐EN at baseline. Follow‐up time was censored at first pregnancy, HIV‐1 seroconversion, last negative STI test during follow‐up or discontinuation of the contraceptive methods of interest. Because only participants from South African sites were exposed to NET‐EN, analyses correlating STI acquisition with NET‐EN use were restricted to South African sites; otherwise, follow‐up was censored at initiation of NET‐EN.

Unadjusted STI incidence rates per 100 women years and exact 95% confidence intervals were estimated by dividing number of post‐baseline STI events by total person years of follow‐up. Contraceptive method specific incidence rates were estimated by dividing number of post‐baseline STI events that occurred while primarily exposed to the contraceptive method divided by the person years of exposure to the primary contraceptive method. Incidence rates for NET‐EN users were estimated only among South African participants. Exact 95% confidence intervals for STI incidence rates were calculated.

The association between hormonal contraception use and risk of STI was estimated using site‐stratified, time‐varying Andersen‐Gill proportional hazards models adjusted for the following confounders that have been associated with STI acquisition in previous studies: age at screening (dichotomized at 25 years), study arm, baseline STI diagnosis, time‐varying indicator of more than one sex partner, time‐varying partner knowledge of study participation and time‐varying condom usage. To account for contraceptive method switching the exposure was treated as time varying. This is implemented by splitting participant records into distinct observations when contraceptive method was switched. For example, a woman who switched from DMPA to IUD would be represented by two observations at the different time points during her study participation. Estimates of hazard rates and standard errors from the Andersen‐Gill model account for correlation between observations in the data. We used last‐value carried forward imputation for missing time‐varying covariates. Copper IUD use was treated as the reference group. Contraceptive method was treated as a time‐varying exposure. Hazard ratios (HR), 95% confidence intervals (CI), and *p* values are reported.

### 2.5 Ethical approval

The ASPIRE study was approved by local regulatory authorities and annually by the Institutional Review Boards at each of the participating sites, and was overseen by the regulatory infrastructure of the U.S. National Institute of Allergy and Infectious Diseases of the National Institutes of Health. Participants provided written informed consent prior to study participation.

## 3 Results

Of the 2629 women enrolled in the ASPIRE, we restricted our analysis to 2264 women (50.2% from South Africa) who used DMPA (n = 1147), implants (n = 692), NET‐EN (n = 438) or copper IUD (n = 541) at any point during follow‐up.

### 3.1 Baseline characteristics

The mean age was 27 years, less than half (43%) of participants were married, and the majority (84%) had completed some secondary schooling (Table 1). Reported number of sexual partners, condom use at last vaginal sex and knowledge of primary partner's other sexual partnerships were similar across women using different contraceptive methods at baseline. The prevalence of STIs at baseline was high: 12.1% for *C. trachomatis,* 4.1% for *N. gonorrhoeae* and 6.7% for *T. vaginalis*.

**Table 1:** Baseline demographic and behavioural characteristics of DMPA, NET‐EN, Implant and Copper IUD users (N = 2264)

Characteristics	N (%)
Age (years)^a^	27 (06.0)
Has primary sex partner	2255 (99.6)
Married	977 (43.2)
Education	
None	22 (01.0)
Primary	333 (14.7)
Secondary or higher	1909 (84.3)
Live births	
0	191 (08.4)
1	768 (33.9)
2	638 (28.2)
3	386 (17.1)
4 or more	281 (12.4)
Alcohol use in past three months^b^	
Daily	16 (00.7)
Twice weekly	114 (05.0)
Weekly	454 (20.1)
None	1646 (72.7)
Primary sex partner has other partners^c^	
Yes	469 (20.7)
No	487 (21.5)
Don't know	1245 (55.0)
More than one partner in the three months prior to enrolment	368 (16.3)
Partner knowledge of study participation	1722 (76.1)
Condom used at last sex act	1278 (56.5)
Primary partner HIV infected^d^	
Yes	27 (1.2)
No	1222 (53.0)
Don't know	1006 (44.4)
Contraceptive method at study entry	
DMPA	1070 (47.3)
Copper IUD	322 (14.2)
NET‐EN	377 (16.7)
Implant	495 (21.9)
Contraceptive method started at study entry	
DMPA	335 (33.6)
Copper IUD	261 (26.2)
NET‐EN	202 (20.3)
Implant	198 (19.9)
Contraceptive method at any time during the study^e^	
DMPA	1147 (40.7)
Copper IUD	541 (19.2)
NET‐EN	438 (15.5)
Implant	692 (24.6)
Baseline STI diagnosis	
*C. trachomatis*	274 (12.1)
*N. gonorrhoeae*	93 (04.1)
*T. vaginalis*	152 (06.7)

DMPA, depot medroxyprogesterone acetate; IUD, intrauterine devices, STI, sexually transmitted infection

^a^Mean (SD) reported; ^b^34 missing; ^c^63 missing; ^d^9 missing; ^e^used any of the listed methods during follow up.

### 3.2 Incidence of STIs by contraceptive method

Over 3440 person‐years of follow‐up, 408 cases of *C. trachomatis,* 196 cases of *N. gonorrhoeae* and 213 cases of *T. vaginalis* were detected, yielding incidence rates of 11.86, 5.70 and 6.19 per 100 person‐years respectively.

The incidence of *C. trachomatis* was 13.32, 6.90, 12.10 and 18.85 per 100 person‐years, among copper IUD, implant, DMPA and NET‐EN users respectively (Table 2). These results were not statistically significantly different in adjusted analyses. Of note, compared to copper IUD, the incidence of C. *trachomatis* was 31% lower among Implant users though this association was just outside of statistical significance. Similarly, the incidence of *N. gonorrhoeae* was 6.46, 5.95, 5.02 and 6.20 per 100 person‐years among copper IUD, implant, DMPA and NET‐EN users respectively, and these differences were not significant in adjusted models.

**Table 2:** Incidence of sexually transmitted infections (STIs) by contraceptive method

Method	*Chlamydia trachomatis*			*Neisseria gonorrhoeae*			*Trichomonas vaginalis*		
	Incidence^a^	aHR^b^	95% CI	Incidence^a^	aHR^b^	95% CI	Incidence^a^	aHR^b^	95% CI
IUD (copper)^c^	13.32	1.00	‐	6.46	1.00	‐	10.15	1.00	‐
Implant	6.90	0.69	0.47,1.01	5.95	0.97	0.60,1.57	6.78	0.58	0.39,0.87
DMPA	12.10	0.86	0.65,1.16	5.02	0.74	0.48,1.14	4.13	0.37	0.24,0.59
NET‐EN^d^	18.85	1.45	0.94,2.23	6.20	0.86	0.48,1.57	4.91	0.40	0.20,0.81

CI, confidence intervals; DMPA, depot medroxyprogesterone acetate; IUD, intrauterine devices

^a^Incidence per 100 person‐years; ^b^hazard ratios adjusted for randomization arm, age, baseline sexual behaviour (number of partners in the last three months, and condom use at last sex act), baseline STIs; ^c^copper IUD; ^d^NET‐EN only South African women.

The incidence of *T. vaginalis* was higher for copper IUD users (10.15 per 100 person‐years) compared to DMPA (6.78 per 100 person‐years), implant (4.13 per 100 person‐years) and NET‐EN users (4.91 per 100 person‐years), and these differences were statistically significant in adjusted models: aHR 0.37 (95% CI 0.24, 0.59) for DMPA, aHR 0.58 (95% CI 0.39, 0.87) for implant; and aHR 0.40 (95% CI 0.20, 0.81) for NET‐EN, each compared to copper IUD. All findings were consistent when separately analysed for South Africa and non‐South Africa sites (data not shown).

## 4 Discussion

In this prospective study of African women at elevated risk for HIV‐1 acquisition, we observed high rates of STIs, with an overall incidence of *C. trachomatis* >10% per year. The risk of cervical infections (*C. trachomatis* and *N. gonorrhoeae*) did not differ significantly across multiple contraceptive methods (DMPA, implants, NET‐EN and copper IUD). On the other hand, copper IUD users had higher incidence of *T. vaginalis* compared to users of progestin‐based methods.

Our finding that progestin based hormonal contraception and copper IUD use were not associated with significantly increased risk of acquisition of cervical chlamydial and gonorrhoeal infections is consistent with some previous studies but not others 15, 16, 17, 19, 23, 24, 25, 26. However, most of these studies have focused on injectable methods 15, 16, 17, 19, 23, 26, many considered non‐users of contraception as the comparison group 16, 19, very few have included copper IUDs 27, and none included implant users. To the best of our knowledge, this is the first study to prospectively investigate the relationship use of progestin based implants and cervical STI acquisition, and is one of a few to do so for the use of copper IUDs. The lower risk of chlamydial infection acquisition among implant users compared to IUD users was nearly statistically significant and could be explored more in future studies to assess whether there is a true association. Furthermore, in contrast to findings of lower risk of *T. vaginalis* among progestin hormonal contraceptive users in our study, a prospective study in Rakai, Uganda found the risk of *T. vaginalis* to be significantly higher among implant users and lower among DMPA users 14. This is likely due to differences in the reference groups namely non‐contraceptive users and copper IUD in the Rakai study and our study respectively.

Higher rates of *T. vaginalis* among IUD users compared to women using progestin based methods may be due to increased menstrual bleeding associated with copper IUD use, differences in vaginal microbiota, and direct or indirect hormone effects. Increased bleeding is a common side effect of copper IUD use, which may increase the growth of trichomonads; *T. vaginalis* growth has been found to be most predominant immediately following menses, when iron availability from lactoferrin is greatest 28. The other possible causal mechanism for the increased risk of *T. vaginalis* among copper IUD is that the changes in microbiota associated with copper use could predispose women to acquiring trichomoniasis. Zimbabwean women using copper IUDs have been reported to have an increase in bacterial vaginosis 29 and studies conducted in populations of women living in Sub Saharan Africa have documented an association between bacterial vaginosis and acquisition of *T. vaginalis* 30. Similarly, large cross‐sectional studies have also reported an association between copper IUD use, bacterial vaginosis and *T. vaginalis compared to non‐contraceptive users* 31 If the association between copper IUD use, bacterial vaginosis and *T. vaginalis* were true, interventions that can cure and/or prevent bacterial vaginosis could be evaluated in clinical trials as a feasible approach to reducing susceptibility to *T. vaginalis* infection among IUD users. In addition, counselling regarding the possible risk of bacterial vaginosis and *T. vaginalis* could also be considered for women choosing copper IUD. Conversely, the alternative explanation may be a protective effect of progestin based contraception against *T. vaginalis* likely due to hypo‐oestrogenic states which may not be conducive for persistence of *T. vaginalis* 23. However, though women using DMPA have suppressed levels of oestrogen 32, 33, 34, the same is not true for NET‐EN or implant users 34. Thus, this hypothesis does not fully explain the consistent finding across all the three progestin contraceptive groups.

Our findings are strengthened by the inclusion of a variety of contraceptive users across multiple geographic locations including implant users, a group that warrants special attention given the increasing uptake of implants in low‐income countries 35. The prospective nature of the study design and the inclusion of over 3440 person‐years of follow‐up allowed for more precise ascertainment of the timing of hormonal contraceptive exposure in relation occurrence of STIs. Additionally, unlike prior studies that used non‐contraceptive users or condom users as the reference group, we compared contraceptive users to each other, with our reference category for adjusted analyses being copper IUD users. Bias related to differences between women who choose to use versus not to use contraception would thus be minimized.

Conversely, this lack of non‐contraceptive users, who would have acted as an additional comparator for the different contraceptive methods precludes our understanding of some of the biological effects of the different contraceptive methods studied. Other study limitations include possible residual confounding related to specific method choice. Given that women chose their own contraceptive method, it is possible that there were some differences in sexual behaviour, and partner characteristics among copper IUD users compared hormonal users that were not captured by our standard behavioural questionnaires. Though such unmeasured confounders may introduce bias in our results, the fact that we found elevated risks for some and not all STIs suggests that residual confounding had minimal influence on our results. Also, since some STI testing was done in response to symptoms, such testing could have amplified associations observed here. In addition, given that disclosure of study participation to male partners was not a requirement for study participation, participants were given an option of bringing in their partners for STI testing and treatment, or not. Unfortunately, information on uptake of partner notification procedures was not captured, and low uptake could have led to re‐infection. However, it's unlikely that partner notification by participants on copper IUD compared to progestin based methods was differential given that all the methods are equally discrete. Furthermore, among women who switched contraceptive methods during the study, we did not account for a “washout” period for switches from Implant, DMPA or NET‐EN to non‐hormonal IUD during study follow‐up. However, we frequently captured data on contraceptive use on a monthly visit throughout the study follow‐up period which minimized possible exposure misclassification bias. We also censored those switched to any method besides copper IUD, Implant, DMPA or NET‐EN (for South African analysis) at the visit they switched. Finally, detection of *T. vaginalis* by OSOM is less sensitive than nucleic acid amplification tests and thus could have resulted in a lower incidence of *T. vaginalis*; however, any misclassification would likely be non‐differential with respect to contraceptive exposure, and would therefore not bias our hazard ratios for the effect of hormonal contraception on *T. vaginalis* acquisition.

## 5 Conclusions

Among African women at high HIV‐1 risk, STIs were common. The risk of cervical infections did not differ across contraceptive methods, although significantly higher rates of *T. vaginalis* were observed among progestin based methods compared to copper IUD users. The high burden of STIs among the different contraceptive users despite counselling and condom distribution provided to participants in this study calls for more intensive routine screening and risk‐reduction behavioural counselling addressing knowledge and practices leading to STI risk reduction, beyond simply reducing unplanned pregnancy.

Women at high risk for STIs should therefore continue to have access to a wide range of contraceptive choices 36, 37 and do not need to be discouraged from using progestin based contraceptives or copper IUDs. Instead, providers should emphasize condom use and other STI prevention strategies for women and their sexual partners.

## Competing interests

The authors have no conflict of interest to declare.

## Authors’ contributions

FMK, ERB, GN, TPP, NM, CN, NC, SLH and JMB performed the research. JMB, TPP, ERB and SLH designed the research study. Data analysis was done by ERB and AM. Data interpretation and manuscript preparation was performed by FMK guided by JMB. All authors contributed to the final version of the manuscript; revised the article critically for important intellectual content and approved the final manuscript.

## Appendix 1 MTN‐020/ASPIRE Study Team

### Study team leadership

Jared Baeten, University of Washington (Protocol Chair); Thesla Palanee‐Phillips, Wits Reproductive Health and HIV Institute (Protocol Co‐chair); Elizabeth Brown, Fred Hutchinson Cancer Research Center (Protocol Statistician); Lydia Soto‐Torres, US National Institute of Allergy and Infectious Diseases (Medical Officer); Katie Schwartz, FHI 360 (Clinical Research Manager).

### Study sites and site investigators of record

Malawi, Blantyre site (Johns Hopkins University, Queen Elizabeth Hospital): Bonus Makanani; Malawi, Lilongwe site (University of North Carolina, Chapel Hill): Francis Martinson, South Africa, Cape Town site (University of Cape Town): Linda‐Gail Bekker; South Africa, Durban – Botha's Hill, Chatsworth, Isipingo, Tongaat, Umkomaas, Verulam sites (South African Medical Research Council): Vaneshree Govender, Samantha Siva, Zakir Gaffoor, Logashvari Naidoo, Arendevi Pather, and Nitesha Jeenarain; South Africa, Durban, eThekwini site (Center for the AIDS Programme for Research in South Africa): Gonasagrie Nair, South Africa, Johannesburg site (Wits Reproductive Health and HIV Institute, University of the Witwatersrand): Thesla Palanee‐Phillips, Uganda, Kampala site (John Hopkins University, Makerere University): Flavia Matovu Kiweewa, Zimbabwe, Chitungwiza, Seke South and Zengeza sites (University of Zimbabwe College of Health Sciences Clinical Trials Research Centre): Nyaradzo Mgodi, Zimbabwe, Harare, Spilhaus site (University of Zimbabwe College of Health Sciences Clinical Trials Research Centre): Felix Mhlanga, Data management was provided by The Statistical Center for HIV/AIDS Research & Prevention (Fred Hutchinson Cancer Research Center, Seattle, WA) and site laboratory oversight was provided by the Microbicide Trials Network Laboratory Center (Pittsburgh, PA).
