# Erratum

**DOI:** 10.1002/jia2.25280

**Published:** 2019-04-15

Regarding the paper “Acquisition of Sexually Transmitted Infections among Women Using a Variety of Contraceptive Options: A prospective Study among High‐risk African Women” by Flavia Matovu Kiweewa, Elizabeth Brown, Anu Mishra, Gonasagrie Nair, Thesla Palanee‐Phillips, Nyaradzo Mgodi, Clemensia Nakabiito, Nahida Chakhtoura, Sharon L Hillier, Jared M Baeten for the MTN‐020/ASPIRE Study Team.

Published in Journal of the International AIDS Society, Citation: Journal of the International AIDS Society 2019, 22: 25257. https://onlinelibrary.wiley.com/doi/10.1002/jia2.25257

There is an error in the abstract (Conclusions) and in the Conclusions section.

‘Significantly higher rates of T. vaginalis was observed among progestin based methods compared to copper IUD users’ should read ‘Significantly higher rates of T. vaginalis were observed among copper IUD compared to progestin hormonal contraceptive users’.

